# Effect of 3`-Azido-3`-Deoxythymidine (AZT) on Telomerase Activity and Proliferation of HO-8910 Cell Line of Ovarian Cancer

**Published:** 2006-02

**Authors:** Jonathan D. Dinman

Though the article of “Effect of 3′-azido-3′-deoxythymidine (AZT) on telomerase activity and proliferation of HO-8910 cell line of ovarian cancer” raises more questions than it answers, it presents an interesting new line of research that has potential for identifying an important drug target for the treatment of cancer. Actively dividing cells, e.g. cancer cells, need to replicate the ends of their telomeres. The polynucleotide polymerase that performs this task, Telomerase, is critical to this function, and thus presents a rational target for anti-cancer therapeutics. Nucleoside analogs have been used to target other polynucleotide polymerases, e.g. retroviral reverse transcriptases. Thus, it is reasonable to conjecture that nucleoside analogs might be applied to this problem as well. There is an emerging literature examining the effects of AZT on cancer cell lines. Though it is clear that this drug can kill cancer cells, the dosages used in these studies are well above any biologically meaningful range, and is not clear that the cytoxic effects are due to direct effects on telomerase activity, or simple general metabolic poisoning of cells. While this emerging field of inquiry may hold some promise, direct biochemical studies comparing rates of incorporation of nucleoside analogs into DNA by a variety of human polynucleotide polymerases (e.g. the various cellular DNA polymerases, RNA polymerases, and telomerase) needs to be investigated. Should a preferred set of substrates be identified for telomerase, this may very well become the foundation for a new class of anti-neoplastic therapies.

